# Meteorin-like/Meteorin-β protects LPS-induced acute lung injury by activating SIRT1-P53-SLC7A11 mediated ferroptosis pathway

**DOI:** 10.1186/s10020-023-00714-6

**Published:** 2023-10-25

**Authors:** Zhen Chen, Jun Li, Huan Peng, Mengli Zhang, Xian Wu, Feng Gui, Wei Li, Fen Ai, Bo Yu, Yijue Liu

**Affiliations:** 1 Department of Emergency, The Central Hospital of Wuhan, Tongji Medical College, Huazhong University of Science and Technology, Wuhan, Hubei Province People’s Republic of China; 2 Department of Critical Care Medicine, The Central Hospital of Wuhan, Tongji Medical College, Huazhong University of Science and Technology, Wuhan, Hubei Province People’s Republic of China

**Keywords:** Metrnβ, Acute lung injury, SIRT1, Ferroptosis, SLC7A11

## Abstract

**Background:**

Ferroptosis plays an essential role in lipopolysaccharide (LPS)-induced acute lung injury (ALI). Meteorin-like/Meteorin-β (Metrnβ) is a protein secreted by skeletal muscle and adipose tissue and plays a role in cardiovascular diseases. However, its role in acute lung injury has not been elucidated.

**Methods:**

In this study, we used an adenovirus (Ad) delivery system to overexpress or knockdown Metrnβ in lung tissue to examine the role of Metrnβ in LPS-induced acute lung injury.

**Results:**

We found that ferroptosis was increased during LPS-induced ALI. The expression of Metrnβ was reduced in ALI lung tissue. Overexpression of Metrnβ in lung tissue alleviated LPS-induced lung injury, inflammation, and ferroptosis. Moreover, Metrnβ knockout in lung tissue accelerated LPS-induced ALI, inflammation, and ferroptosis. We also cultured MLE-12 cells and transfected the cells with Ad-Metrnβ or Metrnβ siRNA. Metrnβ overexpression ameliorated LPS-induced MLE cell death, inflammation and ferroptosis, while Metrnβ knockdown aggregated cell survival and decreased inflammation and ferroptosis. Moreover, we found that Metrnβ enhanced ferroptosis-related Gpx4 expression and reduced ferroportin and ferritin levels. Furthermore, we found that Metrnβ positively regulated SIRT1 transcription thus inhibited P53, increased SLC7A11 expression. When we used the ferroptosis inhibitor ferrostatin-1, the deteriorating effects of Metrnβ knockout were abolished in ALI mice. Moreover, SIRT1 knockout also abolished the protective effects of Metrnβ overexpression in vivo.

**Conclusions:**

Taken together, Metrnβ could protect LPS-induced ALI by activating SIRT1-P53- SLC7A11 mediated ferroptosis inhibition.

## Introduction

Acute lung injury (ALI) is a proinflammatory immune response stimulated by immune recognition of pathogens in lung tissue during severe pulmonary microbial infection (Mokra 2020; Mokra and Kosutova 2015; Butt et al. 2016). Severe acute lung injury can lead to acute respiratory distress syndrome (ARDS) and even death (Butt et al. 2016). When pneumonia occurs, alveolar epithelial cells (AECs) are one of the first-line cells that are attacked by pathogenic microorganisms and recognize pathogens (Nova et al. 2019). During pneumonia, AECs recognize various pathogenic factors, such as lipopolysaccharide (LPS), to activate pathogen-related molecular patterns (PAMPs) or damage-associated molecular patterns (DAMPs), resulting in inflammation, epithelial cell death and lung tissue injury, which lead to pulmonary edema, changes in gas exchange and hypoxemia (Kumar 2020). At present, the treatment of ALI is mainly symptomatic support treatment, focusing on the treatment of basic diseases and bedside care. The development of molecular-based therapies to block the progression of ALI to ARDS is critical to the treatment of ALI.

Ferroptosis is a newly discovered form of cell death that differs from apoptosis and necrosis. It is characterized by iron-dependent lipid peroxidation, and characteristic morphological phenotypes are cell membrane integrity, cell volume reduction, an increase in mitochondrial membrane density, the reduction or even disappearance of mitochondrial cristae, and normal nuclear size (Li et al. 2020a). Ferroptosis is involved in lung diseases (Chen et al. xxxx). The excessive production of redox-active iron in cells promotes the accumulation of reactive oxygen species (ROS) through the Fenton reaction. However, excessive phospholipid hydroperoxide will destroy cell membrane structure and eventually lead to ferroptosis (Li et al. 2020a; Xu et al. 2021). In recent years, ferroptosis has been confirmed to be closely related to intestinal I/R-induced ALI, oleic acid-induced ALI and acute radiation-induced lung injury (RILI) (Li et al. 2020b; Liu et al. 2020; Fan et al. 2021). In addition, a study showed that inhibiting ferroptosis with ferrostatin-1 and p53 apoptosis stimulating protein inhibitor (iASPP) could inhibit the occurrence and development of ALI to protect the lung (Liu et al. 2020; Yin et al. 2021). Therefore, exploring the regulatory mechanism of ferroptosis in ALI may identify new therapeutic targets for ALI.

Meteorin-like/Meteorin-β (Metrnβ), also known as interleukin (IL)-41, is a recently discovered secreted protein that is mainly secreted by skeletal muscle and adipose tissue (Baht et al. 2020). Early studies found that it was mainly involved in the energy metabolism of skeletal muscle and adipose tissue. Recent research found that Metrnβ is closely related to immunity, inflammation, obesity and diabetes (Wu et al. 2020; Ushach et al. 2018). Under isoproterenol stimulation, Metrnβ-deletion mice showed myocardial hypertrophy, fibrosis and heart failure. Metrnβ overexpression could inhibit myocardial remodelling induced by isoproterenol (Ruperez et al. 2021). In addition, a study reported that myocardial cells also secreted Metrnβ and that the concentration of serum Metrn β was correlated with the prognosis of heart failure (Cai et al. 2022). In addition, recent studies have reported that Metrnβ plays an important role in lung diseases. Xun G reported that Meteorin-β/Meteorin like/IL-41 attenuates airway inflammation in house dust mite-induced allergic asthma (Gao et al. 2022). These results indicate the potential role of Metrnβ in ALI. However, the role of Metrnβ in ALI has not been elucidated. In this study, we used an adenovirus (Ad) delivery system to overexpress or knockdown Metrnβ in lung tissue to examine the role and mechanism of Metrnβ in LPS-induced ALI.

## Materials and methods

### Animals and animal model

Male C57BL6J mice aged 8–10 weeks (weight 23.5–27.5 g) were purchased from the Chinese Academy of Medical Sciences & Peking Union Medical College. The animal experiments were performed according to the Guide for the Care and Use of Laboratory Animals published by the US National Institutes of Health (NIH Publication No. 85–23, revised 1996) and were approved by the Animal Care and Use Committee of The Central Hospital of Wuhan, Tongji Medical College, Huazhong University of Science and Technology (HZTJ-20210612). All the animal experiments were conducted in the Experimental Animal Central of Tongji Medical College, Huazhong University of Science and Technology. Each mouse was subjected to LPS injection (L2630, Sigma, USA) (100 μg diluted in 20 μL of sterile normal saline for 48 h) after anesthetization with 3% amobarbital by intraperitoneal injection. Control mice were administered the same volume of normal saline. Six hours after LPS injection, the mice were injected with adenovirus (Ad) harboring Metrnβ or shMetrnβ (60 μl, 5.0–6.5 × 10^13^ GC/ml) via the caudal vein. Forty-eight hours after LPS injection, we flushed the lungs with PBS three times, and the solution was collected. SIRT1-knockout mice (The Jackson Laboratory) were used in the reverse experiment. SIRT1-knockout mice were subjected to LPS stimulation for 48 h to establish the ALI model. In addition, 6 h after LPS injection, SIRT1-knockout mice were injected with Ad-shMetrnβ to knockdown Metrnβ in lung tissue. We administered ferrostatin-1 (Fer-1, 0.8 mg/kg; Sigma‒Aldrich) to the mice 6 h after LPS injection via the caudal vein. All the mice were euthanized by cervical dislocation.

### Histological analysis

The right lung tissues were removed and fixed in 10% formalin, embedded in paraffin and cut into 25 μm sections. H&E staining was performed. Then, a digital camera (Olympus BX 53 microscope, Tokyo, Japan) was used for observation.

### Determination of pulmonary edema

The left lung was collected and weighed to determine the wet weight. The dry weight was recorded after the lung was baked at 80 °C for 48 h. The W/D weight ratio was calculated with the following formula: wet weight/dry weight *100%.

### Cell culture and treatment

MLE12 cells were acquired from the Cell Bank of the Chinese Academy of Sciences (Shanghai, China) and grown in an incubator at 37 °C and 5% CO_2_ in Dulbecco’s modified Eagle’s medium (11965, Gibco, USA) with 10% fetal bovine serum (0500, Gibco, USA), penicillin (100 IU/mL), and streptomycin sulfate (100 μg/mL). The cells were stimulated with LPS (10 ng/µL). The cells were transfected with Metrnβ siRNA or Ad-Metrnβ to knockdown or overexpress Metrnβ (Ribo, Bio., Shanghai, China) with Lipo2000TM transfection reagent (Beyotime, Shanghai, China). Fer-1 (100 nM), which is a specific inhibitor, was administered 6 h after LPS stimulation. Cells were treated with z-VAD-fmk (10 mM, Medchemexpress, USA) to inhibit apoptosis.

### ELISA

The levels of proinflammatory cytokines in the bronchoalveolar lavage fluid of ALI mice and cell lysates were measured with commercially available enzyme-linked immunosorbent assay (ELISA) kits to quantify TNF-α, IL-1 and IL-6 according to the manufacturer’s protocols. Optical density was measured at 450 nm on an ELISA plate reader (Synergy HT, Biotek, Vermont, USA).

### Malondialdehyde (MDA) and GSH levels

MDA levels were detected by a commercial kit (Beyotime, Bio., Shanghai, China). GSH was evaluated with a total GSH assay kit (BC1175, Solarbio, China) according to the kit guidelines. In brief, lung tissues or cells were rinsed in chilled PBS before being homogenized on ice in solution 1, followed by centrifugation at 8000 × *g* for 10 min at 4 °C to retrieve the supernatant for the GSH assay. Then, the samples were incubated at room temperature for 2 min with 140 µL of solution 2 and solution 3, along with standards. The absorbance was read at 412 nm with a microplate reader. Finally, GSH levels were calculated according to the standard curve and normalized to the protein levels, which were quantified by a Bradford protein assay.

### Fe assays

Intracellular, total, ferric, and ferrous Fe levels were determined with an Fe assay kit (ab83366, Abcam) according to the operational guidelines. In brief, lung tissues or cells were rinsed in chilled PBS before being homogenized on ice in Fe assay buffer with a Dounce homogenizer, followed by centrifugation at 16,000 × *g* for 10 min to collect the supernatant for the Fe assay. The samples were then diluted to 100 µL in assay buffer before being incubated with 5 µL of Fe reducer (for total Fe) or assay buffer (for ferrous Fe) and standards at 37 °C for 30 min. Then, 100 µL of the Fe probe was added to each well, mixed, and incubated at 37 °C without light for 1 h. The absorbance was read at 593 nm with a microplate reader. Finally, Fe levels were calculated according to the standard curve and normalized to protein levels, which were quantified by the Bradford protein assay.

### RT‒PCR and western blotting

We used TRIzol reagent to isolate total mRNA. We used a SmartSpec Plus Spectrophotometer (Bio-Rad) to determine mRNA purity with the OD260/OD280 ratios. A total of 2 μg of mRNA was reverse transcribed into cDNA with a cDNA Synthesis Kit (Roche Diagnostics). We used a LightCycler 480 SYBR Green I Master kit (Roche Diagnostics) for amplification. We used GAPDH as a reference.

For western blotting, we used radioimmunoprecipitation (RIPA) lysis buffer to lyse the cells. Total protein concentration was determined by the BCA method, and the samples were then loaded onto SDS‒PAGE gels. After the proteins were transferred to polyvinylidene difluoride (PVDF) membranes (Millipore), they were incubated with primary antibodies at 4 °C overnight. Primary antibodies against Gpx4 (ab125066), Metrnβ (ab235775), SIRT1 (ab110304), P53 (ab26), Ferroportin (ab239511), Ferritin (ab75973), and SLC7A11 (ab175186) were purchased from Abcam (1:100 dilution, Hercules, CA, USA). The blots were developed with enhanced chemiluminescence (ECL) reagents (Bio-Rad, Hercules, CA, USA) and captured by a ChemiDoc MP Imaging System (Bio-Rad). We used β-actin as a reference.

### Dual luciferase reporter assay

GenePharma Inc. (Shanghai, China) helped us construct the SIRT1 3'-UTR luciferase reporter gene plasmid. We generated pSIRT1-WT and pSIRT1-Mut plasmids by subcloning downstream of the luciferase vector. MLE-12 cells were cotransfected with pSIRT1-WT and Metrnβ siRNA or pSIRT1-Mut and Metrnβ siRNA. After incubation, we used a Dual Luciferase Reporter Gene Assay Kit (Beyotime, China) to detect the luciferase activity according to the manufacturer's instructions.

### Chip assay

MLE-12 cell nuclei were isolated using sucrose gradient ultracentrifugation. Briefly, the cells were cross-linked in 1% PFA with protease/phosphatase inhibitors for 10 min and then lysed in ice-cold lysis buffer as reported in a previous study using serial Dounce homogenization and filtration to release the nuclei (Kim et al. 2020). Crude lysate in 2.1 M sucrose solution was layered onto a 2.2 M sucrose bed and centrifuged at 15,000 × *g* for 1 h at 4 °C. The nuclear pellet was used for ChIP assays using a Magna HiSens ChIP kit (Millipore, 17-10460). Chromatin was sheared using a Biorupter (Diagenode, UCD-200) and pulled down with the following antibodies: rabbit IgG (Millipore, CS200581), Metrnβ (Bethyl Labs, A301-985A), and Pol-II (Millipore, 05-623). The resulting ChIP DNA was assayed by qPCR in duplicate for target gene promoter occupancy. ChIP‒qPCR data were analyzed using the ΔΔCt method to obtain fold enrichment relative to IgG.

### Data analysis

ALI data are expressed as the mean ± SD. SPSS 23.3 was used to analyze the data. When comparing data between two groups, Student’s t test was used. When comparing data among four groups, one-way ANOVA followed by Tukey's after hoc test was used. We defined significant differences with p values less than 0.05.

## Results

### Metrnβ was decreased in LPS-induced ALI lung tissue

**Fig. 1:**
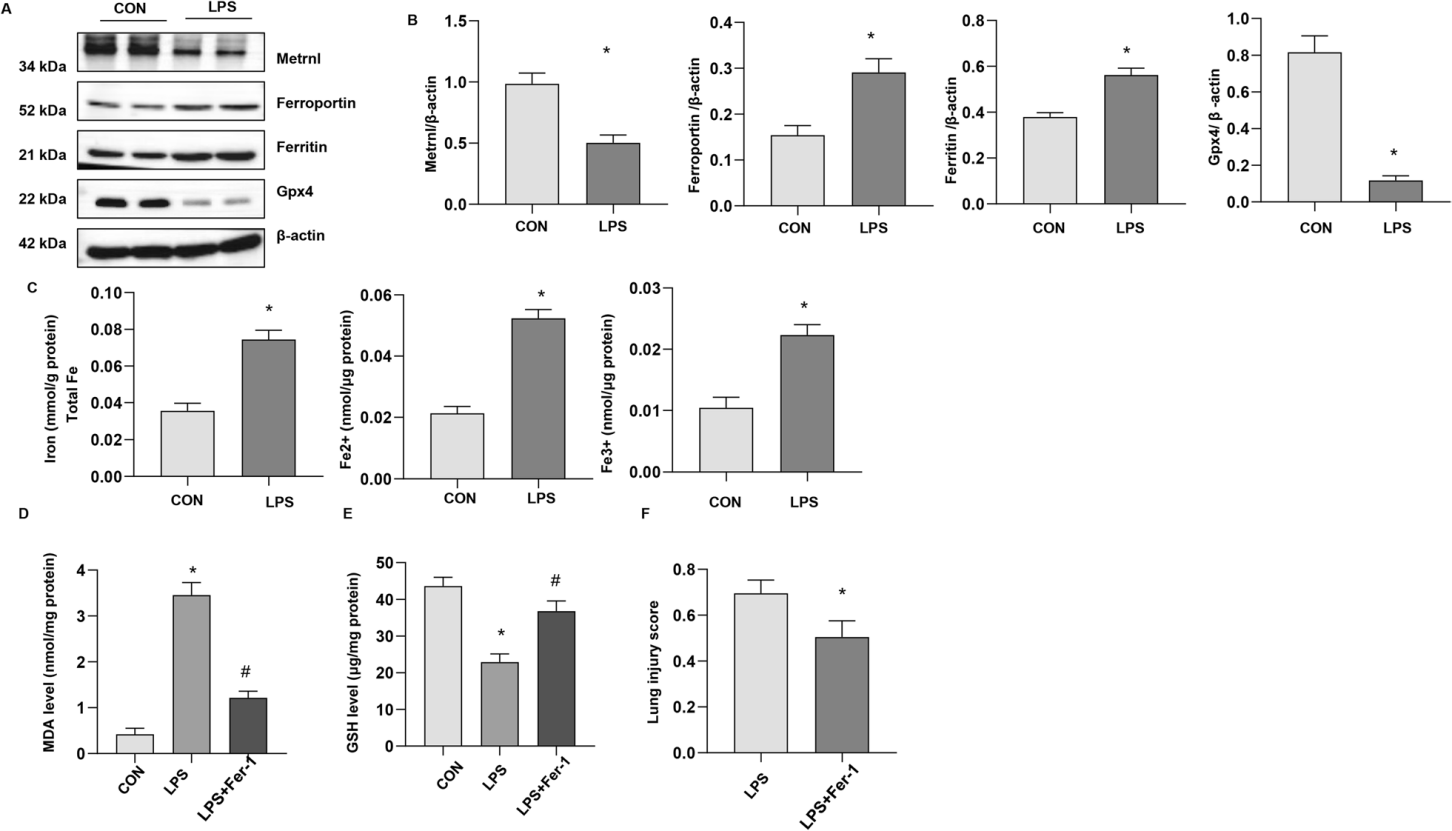
Metrnβ was decreased in LPS-induced ALI lung tissue. **A** and **B** Protein levels of Metrnβ, Ferroportin, Ferritin, and Gpx4 in LPS-induced mouse lungs (n = 4). C. Levels of total iron, Fe^2+^, and Fe3 + in LPS-induced lung tissue (n = 6). Mice were treated with Fer-1. D. MDA levels in LPS-induced lung tissue treated with Fer (n = 6). E. GSH levels in LPS-induced lung tissue treated with Fer (n = 6). F. Lung injury scores in LPS-stimulated mice treated with Fer (n = 6). *P < 0.05 vs. the control group. #P < 0.05 vs. the LPS group

We first evaluated the levels of Metrnβ and ferroptosis in LPS-induced ALI. As shown in Fig. 1a and b, the level of Metrnβ was decreased, and the levels of ferritin and ferroportin (critical ferroptosis proteins) were strongly elevated, while Gpx4 (a critical enzyme regulating lipid peroxidation) was decreased in ALI lung tissue. We also examined endogenous ferric and ferrous levels in the lung tissues in the ALI model. Compared to those in the control group (CON), total Fe, Fe2 + , and Fe3 + levels were markedly increased (Fig. 1c). Moreover, the levels of the lipid peroxide MDA increased, glutathione (GSH) levels were reduced, and the lung injury score was increased (Fig. 1d–f). We used Fer-1, a specific inhibitor, and found that MDA levels were reduced, GSH levels were increased and lung injury scores were decreased (Fig. 1d–f). These data suggested that Metrnβ was reduced and ferroptosis was increased in LPS-induced ALI.

### Metrnβ overexpression ameliorated LPS-induced ALI and ferroptosis

**Fig. 2:**
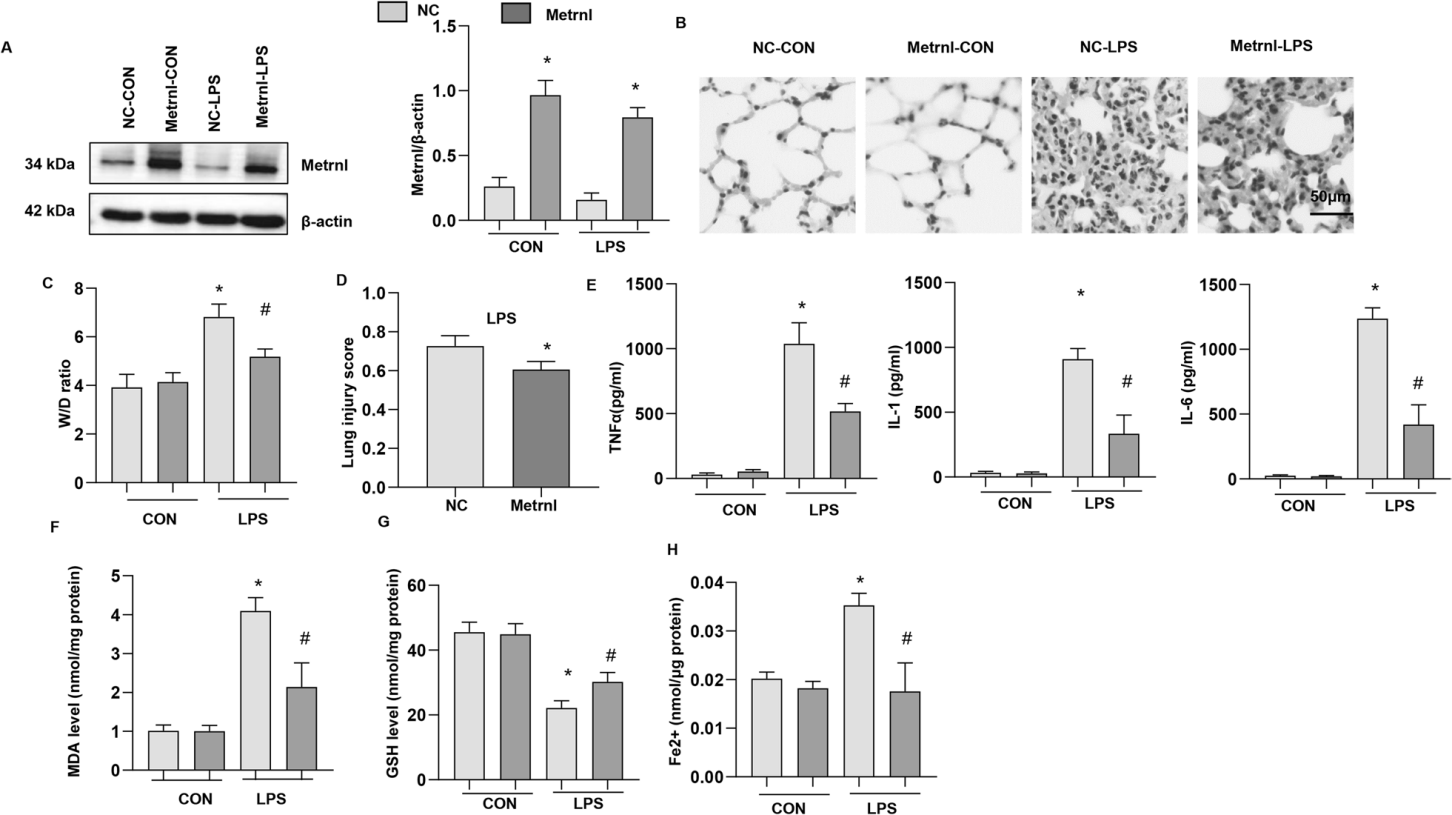
Metrnβ overexpression ameliorated LPS-induced ALI and ferroptosis. **A** Protein level of Metrnβ in mice treated with Ad-Metrnβ and challenged with LPS (n = 4). **B** H&E staining of lung tissue after LPS challenge (n = 6). **C** Lung wet and dry weight ratio (W/D ratio) in mice challenged with LPS (n = 6). **D** Lung injury score in mice challenged with LPS (n = 6). **E** Proinflammatory markers in lung tissue after LPS challenge (n = 6). **F** MDA levels in LPS-induced lung tissue (n = 6). **G** GSH levels in LPS-induced lung tissue (n = 6). **H** Fe^2+^ levels in LPS-induced mouse lung tissue (n = 6). *P < 0.05 vs. the NC-CON group. #P < 0.05 vs. the NC-LPS group

To examine the role of Metrnβ in ALI, we overexpressed Metrnβ in lung tissue (Fig. 2a). LPS induced severe lung injury, as assessed by increases in the lung wet/dry weight ratio and lung injury score and HE staining (Fig. 2b–d), while Metrnβ overexpress reduced lung injury. We also examined inflammatory cytokine release by ELISA and found that LPS sharply increased the release of TNF-a, IL-1 and IL-6, while Metrnβ ameliorated the release of these proinflammatory cytokines (Fig. 2e). Ferroptosis was evaluated (Fig. 2f–h), and LPS increased MDA levels and Fe2 + levels and reduced GSH levels in lung tissue, while Metrnβameliorated these changes. These data indicated that Metrnβcould inhibit LPS-induced ALI and decrease disease severity.

### Metrnβ knockdown in lung tissue exacerbated LPS-induced ALI and ferroptosis in mice

**Fig. 3:**
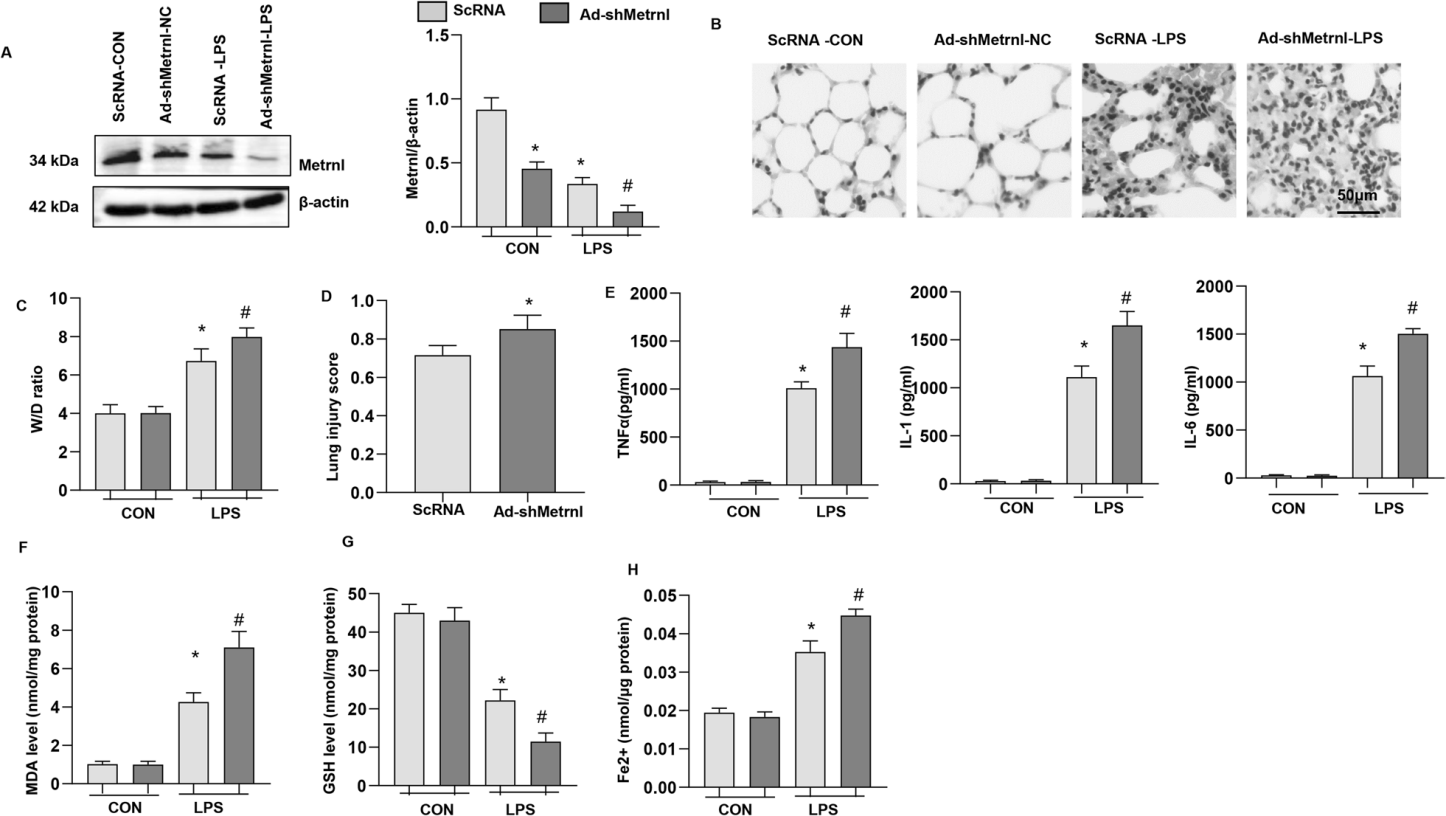
Metrnβ knockdown in lung tissue exacerbated LPS-induced ALI and ferroptosis in mice **A** Protein level of Metrnβ in mice treated with Ad-shMetrnβ and challenged with LPS (n = 4). **B** H&E staining of lung tissue after LPS challenge (n = 6). **C** Lung wet and dry weight ratio (W/D ratio) in mice challenged with LPS (n = 6). **D** Lung injury score in mice challenged with LPS (n = 6). **E** Levels of proinflammatory markers in lung tissue after LPS challenge (n = 6). **F** MDA levels in LPS-induced lung tissue (n = 6). **G** GSH levels in LPS-induced lung tissue (n = 6). **H** Fe^2+^ levels in LPS-induced mouse lung tissue (n = 6). *P < 0.05 vs. the Ad-ScRNA-CON group. #P < 0.05 vs. the Ad-ScRNA-LPS group

To confirm the role of Metrnβ in LPS-induced ALI, we knocked down Metrnβ in lung tissue by adenovirus injection. As shown in Fig. 3a, the level of Metrnβ was markedly decreased in lung tissue after Ad-Metrnβ injection. LPS-induced lung injury was further enhanced by Metrnβ knockdown, as evidenced by the increased W/D ratio and lung injury score in the Ad-shMetrnβ group compared with the Ad-ScRNA group (Fig. 3b–d). The inflammatory response induced by LPS was also exacerbated in the Ad-shMetrnβ group compared with the Ad-ScRNA group (Fig. 3e). LPS-induced ferroptosis was exacerbated by Ad-shMetrnβ injection compared with Ad-ScRNA injection, as evidenced by the levels of MDA, Fe2 + and GSH (Fig. 3f–h). Taken together, these results suggest the protective role of Metrnβ in LPS-induced ALI.

### Ferroptosis increased in LPS-stimulated MLECs

**Fig. 4:**
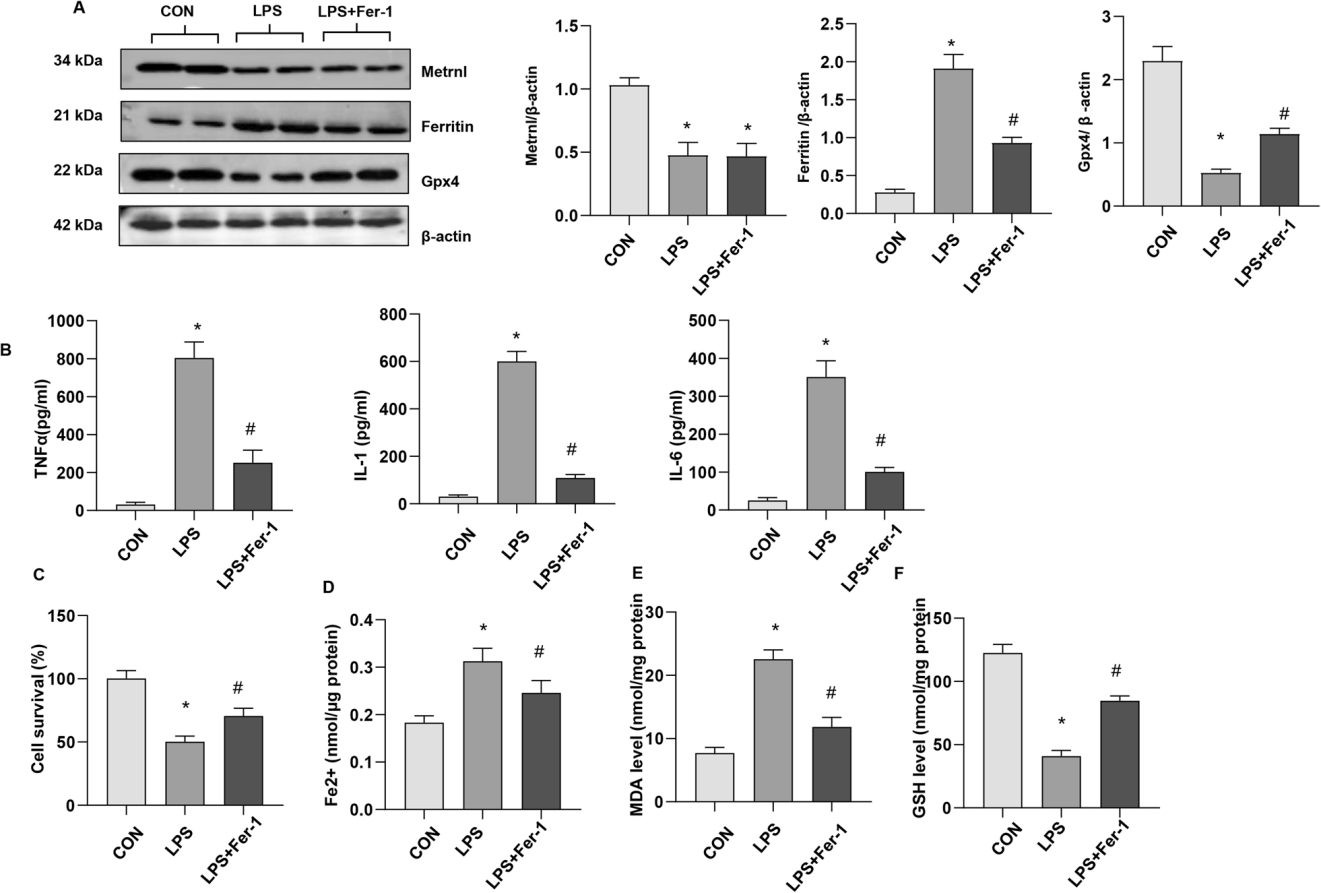
Ferroptosis increased in LPS-stimulated MLECs. MLE-12 cells were treated with LPS and Fer-1. **A** Protein levels of Metrnβ, Ferritin, and Gpx4 in LPS-stimulated MLECs (n = 4). **B** Levels of proinflammatory markers in MLECs treated with LPS and/or Fer-1 (n = 6). **C** The viability of MLECs treated with LPS and/or Fer-1 (n = 6). **D** Fe^2+^ levels in MLECs treated with LPS and/or Fer-1 (n = 6). **E** MDA levels in MLECs treated with LPS and/or Fer-1 (n = 6). **F** GSH levels in MLECs treated with LPS and/or Fer-1 (n = 6). *P < 0.05 vs. the control group. #P < 0.05 vs. the LPS group

Alveolar epithelial cells are primarily involved in damage or danger-associated molecular patterns (DAMPs), which cause lung injury and inflammation. We assessed the ferroptosis level in MLE-12 cells. As shown in Fig. 4a, LPS induced high expression of Ferritin and reduced expression of Gpx4 was ameliorated by Fer-1, a specific inhibitor of ferroptosis. The reduced expression of Metrnβ by LPS insult was not rescued by Fer-1 treatment (Fig. 4a). LPS increased the inflammatory response, as assessed by TNF-a, IL-1, and IL-6 release, while Fer-1 reduced proinflammatory cytokine release (Fig. 4b). LPS stimulation reduced cell viability, increased Fe2 + and MDA levels, and reduced GSH levels. Fer-1 increased cell viability, decreased Fe2 + and MDA levels, and enhanced GSH levels (Fig. 4c-f). These results suggest that ferroptosis occurred in LPS-stimulated AECs and that inhibiting ferroptosis could ameliorate LPS-induced cell injury.

### Metrnβ overexpression ameliorated LPS-induced MLE-12 cell injury and ferroptosis

**Fig. 5:**
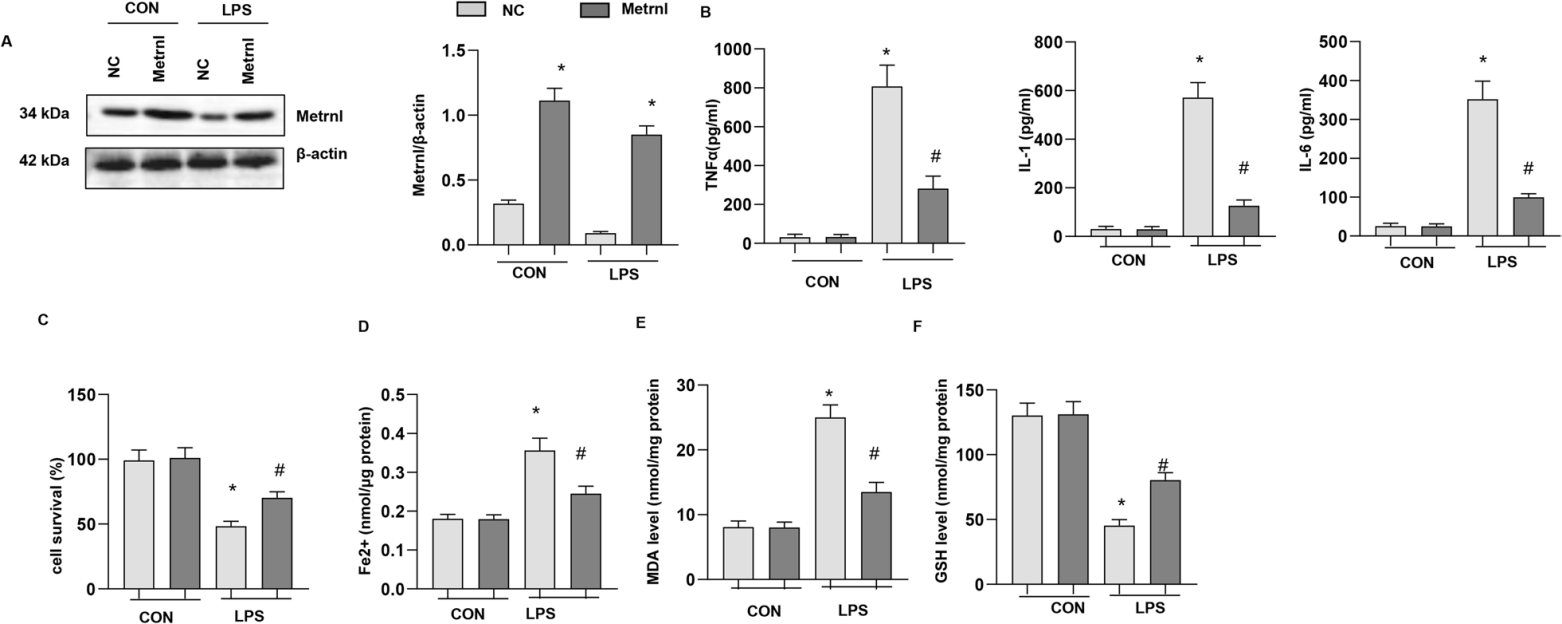
Metrnβ overexpression ameliorated LPS-induced MLE-12 cell injury and ferroptosis. MLE-12 cells were transfected with Ad-Metrnβ and treated with LPS. **A** Protein levels of Metrnβ after Ad-Metrnβ transfection (n = 4). **B** Levels of proinflammatory markers in MLECs (n = 6). **C** The viability of MLECs (n = 6). **D** Fe^2+^ levels in MLECs (n = 6). **E** MDA levels in MLECs (n = 6). **F** GSH levels in MLECs (n = 6). *P < 0.05 vs. the NC-CON group. #P < 0.05 vs. the NC-LPS group

To confirm the role of Metrnβ in AECs, we transfected MLE-12 cells with Ad-Metrnβ (Fig. 5a). Metrnβ overexpression in MLE-12 cells inhibited LPS-induced inflammatory cytokine release (Fig. 5b), increased cell viability (Fig. 5c), decreased Fe2 + and MDA levels (Fig. 5d, e) and increased GSH levels (Fig. 5f).

### Metrnβ knockdown exacerbated LPS-induced MLE-12 cell injury and ferroptosis

**Fig. 6:**
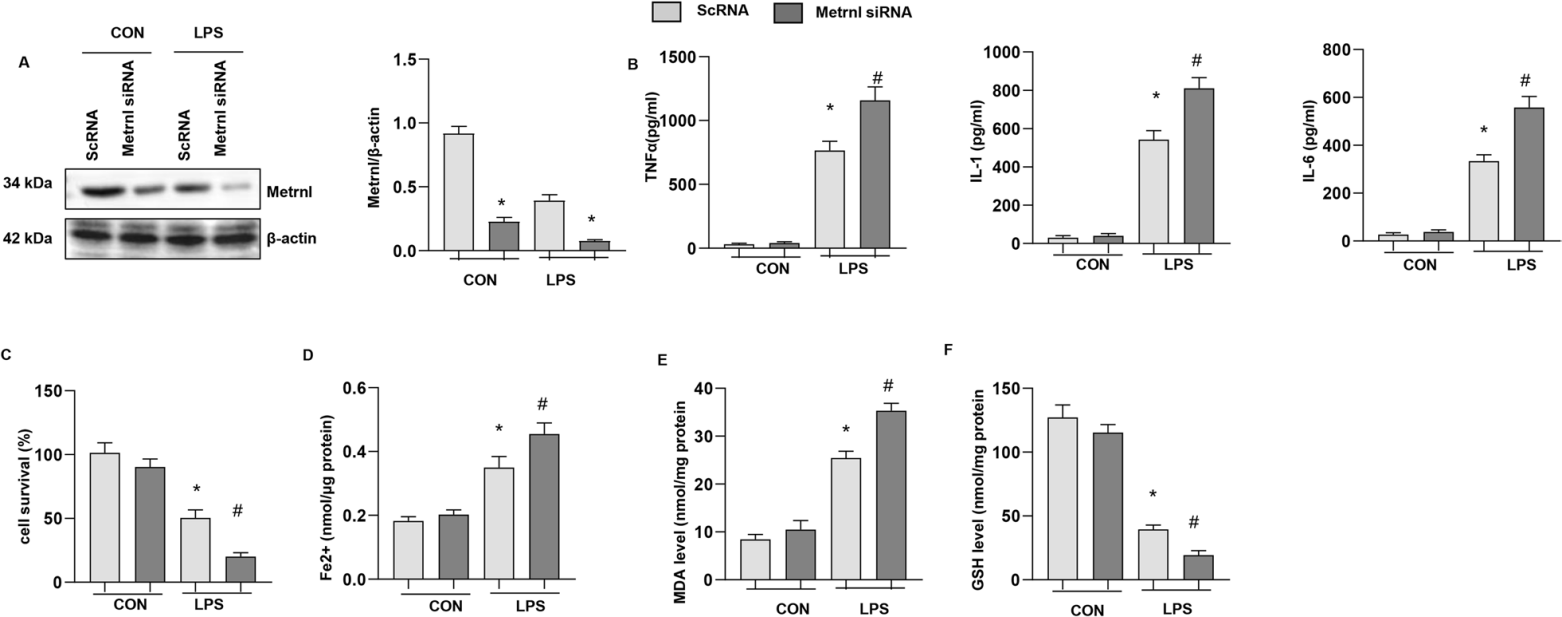
Metrnβ knockdown exacerbated LPS-induced MLE-12 cell injury and ferroptosis. MLE-12 cells were transfected with Metrnβ siRNA and treated with LPS. **A** Protein levels of Metrnβ after Metrnβ siRNA transfection (n = 4). **B** Levels of proinflammatory markers in MLECs (n = 6). **C** The viability of MLECs (n = 6). **D** Fe^2+^ levels in MLECs (n = 6). **E** MDA levels in MLECs (n = 6). **F** GSH levels in MLECs (n = 6). *P < 0.05 vs. the ScRNA-CON group. #P < 0.05 vs. the ScRNA-LPS group

We transfected MLE-12 cells with Metrnβ siRNA to knockdown Metrnβ. As shown in Fig. 6a, the protein level of Metrnβ was reduced after Metrnβ siRNA transfection. LPS-induced cell inflammation was further enhanced by Metrnβ siRNA, as indicated by increased TNF-a, IL-1, and IL-6 levels in MLE-12 cells in the Metrnβ siRNA group compared with those in the ScRNA group (Fig. 6b). LPS-induced ferroptosis was also exacerbated by Metrnβ knockdown in MLE-12 cells, as indicated by reduced cell viability, increased MDA and Fe2 + levels, and decreased GSH levels in the Metrnβ siRNA g group compared with the ScRNA group (Fig. 6c–f).

### Metrnβ regulated the SIRT1-SLC7A11 pathway to regulate ferroptosis

**Fig. 7:**
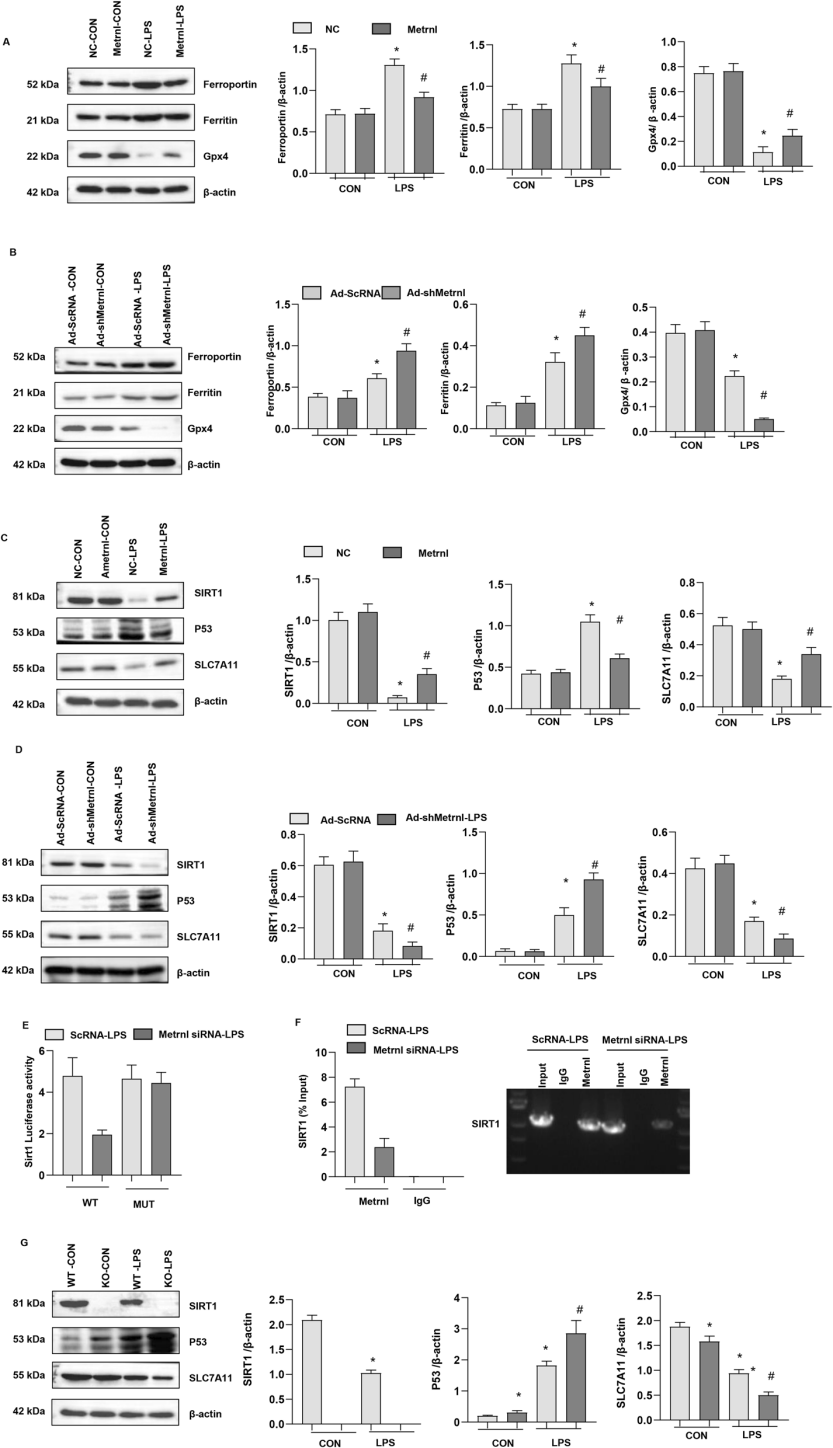
Metrnβ regulated the SIRT1-P53-SLC7A11 pathway to regulate ferroptosis. **A** Protein levels of Ferroportin, Ferritin, and Gpx4 in mice lung tissue treated with Ad-Metrnβ and challenged with LPS (n = 4). *P < 0.05 vs. the NC-CON group. #P < 0.05 vs. the NC-LPS group. **B** Protein levels of Ferroportin, Ferritin, and Gpx4 in mice lung tissue treated with Ad-shMetrnβ and challenged with LPS (n = 4). *P < 0.05 vs. the Ad-ScRNA-CON group. #P < 0.05 vs. the Ad-ScRNA-LPS group. **C** Protein levels of SIRT1, P53, and SLC7A11 in mice treated with Ad-Metrnβ and challenged with LPS (n = 4). *P < 0.05 vs. the NC-CON group. #P < 0.05 vs. the NC-LPS group. **D** Protein levels of SIRT1, P53, and SLC7A11 in mice lung tissue treated with Ad-shMetrnβ and challenged with LPS (n = 4). *P < 0.05 vs. the Ad-ScRNA-CON group. #P < 0.05 vs. the Ad-ScRNA-LPS group. **E** Luciferase analysis of MLE-12 cells co-transfected with pSIRT1-WT and Metrnβ siRNA or pSIRT1-Mut and Metrnβ siRNA challenged with LPS. *P < 0.05 vs. the ScRNA group. **F** ChIP analysis of MLE-12 cells challenged with LPS cultured with Metrnβ antibody. **G** Protein levels of SIRT1, P53, and SLC7A11 in mice lung tissue challenged with LPS (n = 4). *P < 0.05 vs. theWT-CON group; #P < 0.05 vs. the WT-LPS group

To confirm the role of Metrnβ in ferroptosis in LPS-induced ALI in vivo and in vitro, we measured the levels of ferroptosis-associated proteins. As shown in Fig. 7a, the levels of ferroportin and ferritin were elevated, but Gpx4 was reduced in the two LPS groups. Lung tissue in the Ad-Metrnβ group showed reduced ferroportin and ferritin levels but increased Gpx4 levels compared with that in the Ad-NC group under LPS stimulation. In lung tissue in Ad-shMetrnβ group, we also observed increased ferroportin and ferritin levels and reduced Gpx4 levels in the LPS group compared with the control group (Fig. 7b). Metrnβ knockdown enhanced ferroportin and ferritin levels and decreased Gpx4 levels compared to those in the ScRNA group under LPS stimulation (Fig. 7b).

A previous study reported that Metrnβ affects mitochondrial proteins. And previous study reported that Metrnβ could activate SIRT1 in cardiomyocytes [20. We examined mitochondrial-associated proteins and found that SIRT1, which regulates mitochondrial function and redox metabolism, was reduced in LPS-stimulated lung tissue (Fig. 7c). P53, the downstream target of SIRT1, was increased in LPS-stimulated lung tissue. SLC7A11, a transcription factor that inhibits ferroptosis, was also reduced in the LPS group (Fig. 7c). Metrnβ overexpression increased SIRT1 levels, reduced P53 expression and enhanced SLC7A11 protein levels in lung tissue in the LPS group (Fig. 7c). While Metrnβ knockdown decreased SIRT1 levels, enhanced P53 expression and inhibited SLC7A11 expression. To evaluate whether Metrnβ could directly regulate SIRT1 transcription levels, a luciferase assay was performed, and the results are shown in Fig. 7e. Metrnβ silence inhibited the promoter activity of SIRT1. We also performed a ChIP assay to confirm the regulatory effect of Metrnβ on SIRT1. As shown in Fig. 7f, Metrnβ bound to the promoter region of SIRT1. To further confirm SIRT1 regulate P53 and SLC7A11 signaling, we used SIRT1-KO mouse, and found that P53 was up-regulated and SLC7A11 was down-regulated in SIRT1-KO lung tissue in both normal condition and LPS-insult (Fig. 7g). These data indicate that Metrnβ regulates LPS-induced ferroptosis by regulating SIRT1-P53-SLC7A11 signaling.

### The protective effects of Metrnβ on ALI is independent of apoptosis inhibition

**Fig. 8:**
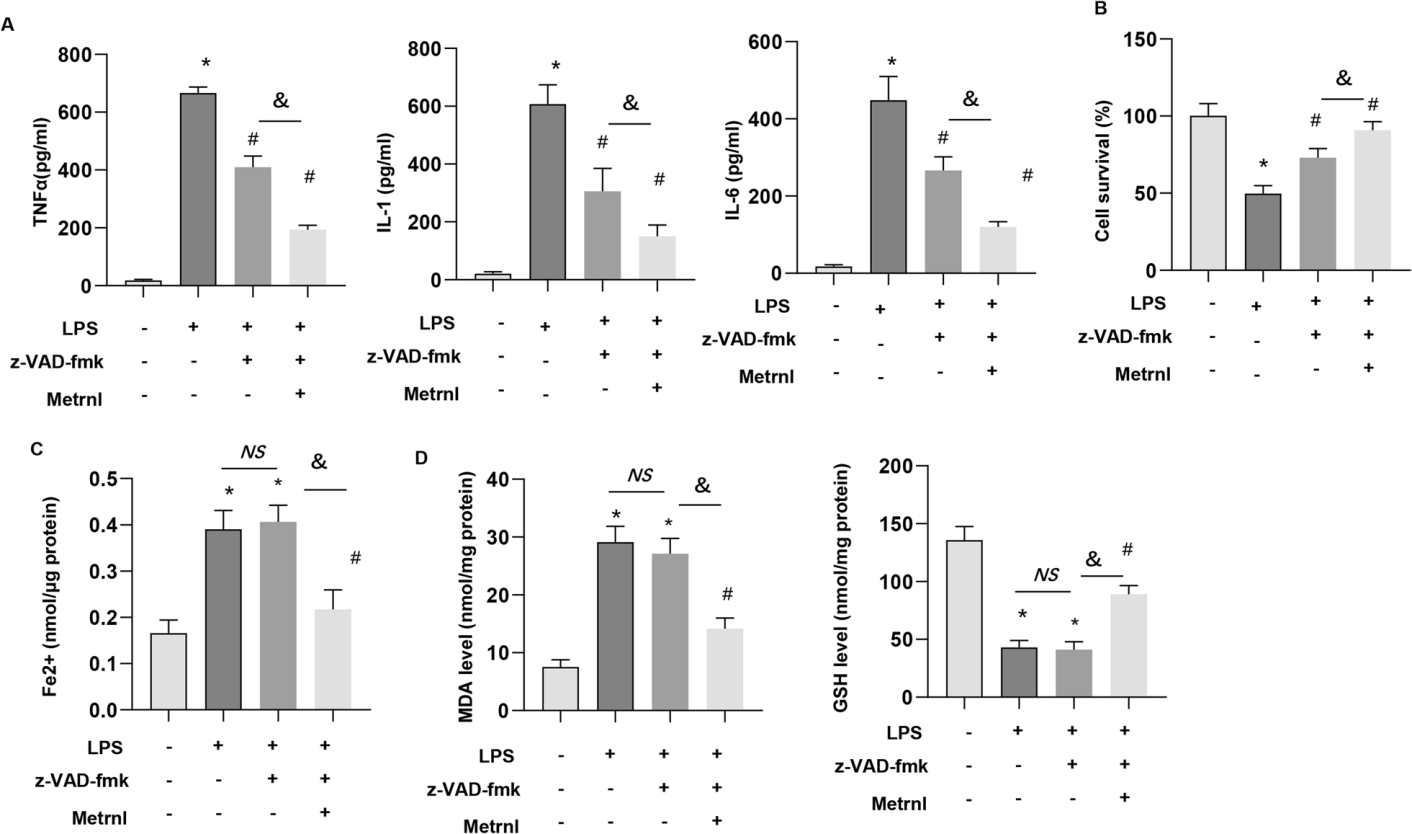
Ferroptosis inhibition or activation abolished Metrnβ-mediated effects in vitro. MLE-12 cells were transfected with Ad-Metrnβ or treated with VAD-fmk (10 mM). **A** Levels of proinflammatory markers in MLECs (n = 6). **B** The viability of MLECs (n = 6). **C** Fe^2+^ levels in MLECs (n = 6). **D** MDA levels in MLECs (n = 6). **F** GSH levels in MLECs (n = 6). *P < 0.05 vs. the CON group, #P < 0.05 vs. the LPS group. ^**&**^P < 0.05. NS: no significant

To rule out the effects of Metrnβ on apoptosis, cells were treated with apoptosis inhibitor z-VAD-fmk. As shown in Fig. 8, LPS-induced inflammation response was suppressed by z-VAD-fmk, and the cell viability was also enhanced by z-VAD-fmk treatment (Fig. 8a–d). While ferroptosis was not suppressed in z-VAD-fmk treated cells as evidenced by Fe^2+^ level, MDA level and GSH level (Fig. 8a–d). Cells in z-VAD-fmk and Ad-Metrnβ treatment group showed ameliorated inflammation response, cell viability and ferroptosis as compared with cells treated with z-VAD-fmk only. Thus, our data suggest Metrnβ protects against ALI independent of apoptosis inhibition.

### Ferroptosis inhibition or activation abolished Metrnβ-mediated effects in vitro

**Fig. 9:**
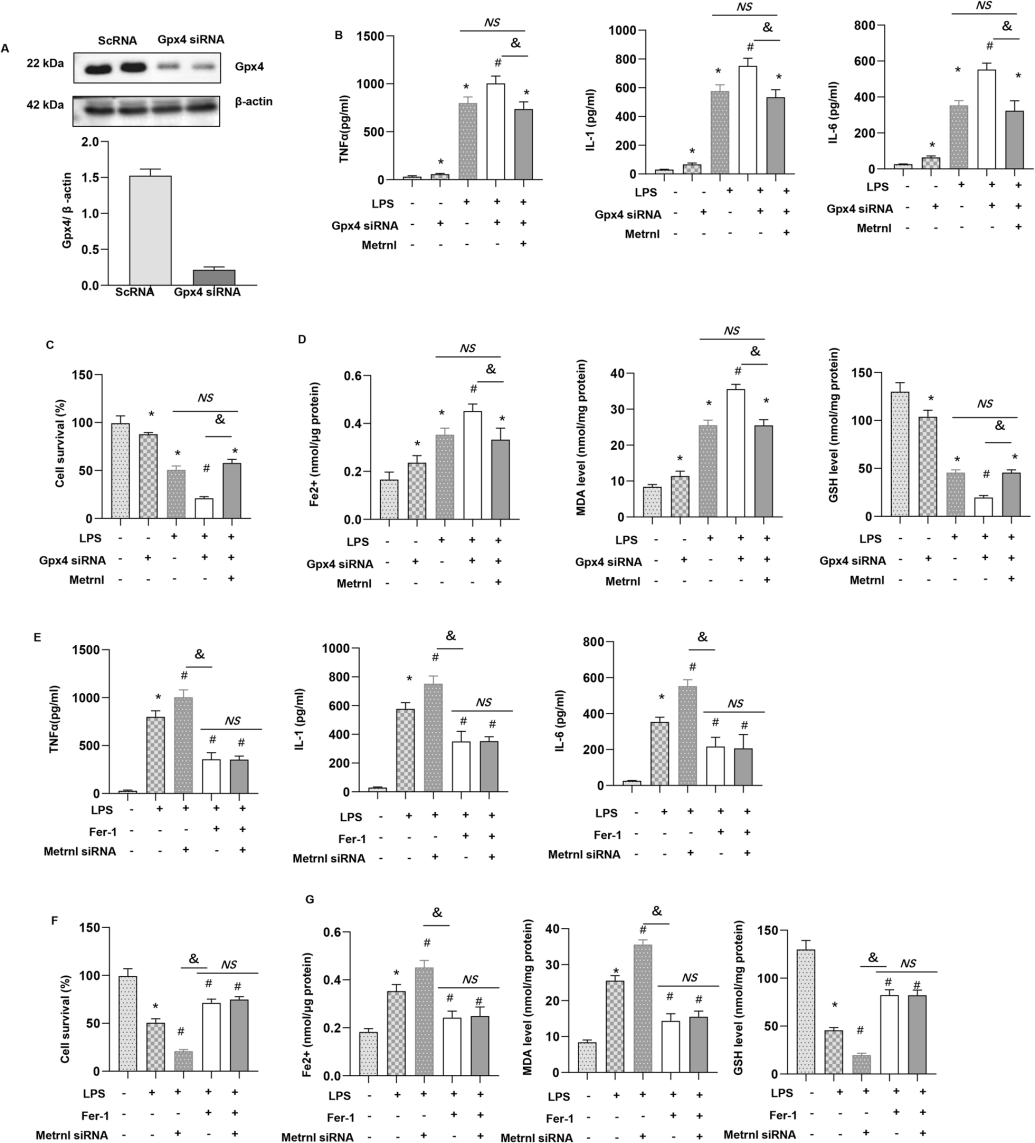
Ferroptosis inhibition or activation abolished Metrnβ-mediated effects in vitro. **A**–**D** MLE-12 cells were transfected with Ad-Metrnβ and/or cotransfected with Gpx4 siRN**A** **A** Protein levels of Gpx4 after the cells were transfected with Gpx4 siRNA (n = 4). **B** Levels of proinflammatory markers in MLECs (n = 6). **C** The viability of MLECs (n = 6). **D** Fe^2+^ levels in MLECs (n = 6); MDA levels in MLECs (n = 6); GSH levels in MLECs (n = 6). *P < 0.05 vs. the CON group; #P < 0.05 vs. the LPS group. ^**&**^P < 0.05. NS: no significant. E–G: MLE-12 cells were transfected with Metrnβ siRNA and/or treated with Fer-1. **E** Levels of proinflammatory markers in MLECs (n = 6). **F** The viability of MLECs (n = 6). **G** Fe^2+^ levels, MDA levels, GSH levels in MLECs (n = 6). *P < 0.05 vs. the CON group; #P < 0.05 vs. the LPS group. ^**&**^P < 0.05. *NS* no significant

To confirm that ferroptosis is the target of Metrnβ in ALI, we transfected MLE-12 cells with Gpx4 siRNA to knockdown Gpx4. Then treated cells with Ad-Metrnβ. Gpx4 siRNA reduced the protein level of Gpx4 (Fig. 9a). Gpx4 knockdown caused deteriorated inflammation and ferroptosis in both normal condition and LPS challenge. Metrnβ could at least partly counteracted the deteriorating effects of Gpx4 knockdown in ALI invitro (MDA, Fe2 + and GSH levels, cell viability) (Fig. 9b–d). However, the inflammation and ferroptosis levels were comparable in Gpx4 siRNA + Ad-Metrnβ group and LPS group (Fig. 9b–d). We also used Fer-1 to further confirm the effects of Metrnβ on MEL-12 cells. Metrnβ silence caused detrimental effects on MEL-12 cells under LPS stimulation that were abolished by Fer-1, as indicated by the amelioration of inflammation and ferroptosis (MDA, Fe2 + and GSH levels, cell viability) (Fig. 9e–g). However, the inflammation and ferroptosis levels were comparable in Fer-1 + LPS group and Fer-1 + Metrnβ siRNA + LPS group (Fig. 9e–g). These data suggest that Metrnβ protects LPS-induced ALI by inhibiting ferroptosis in AECs.

### Fer-1 counteracted the effects of Metrnβ knockdown in vivo

**Fig. 10:**
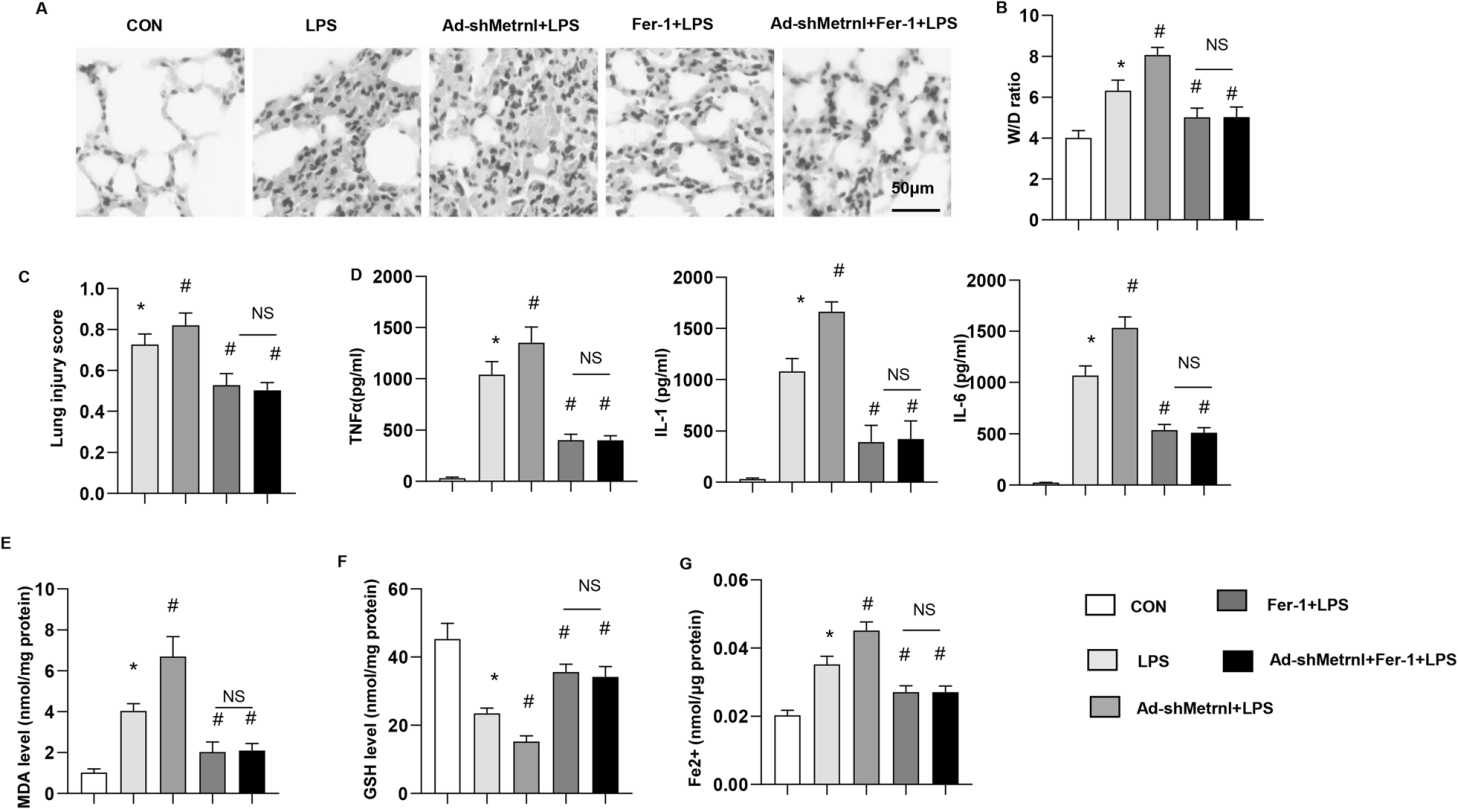
Fer-1 counteracted the effects of Metrnβ knockdown in vivo. Mice were treated with Ad-shMetrnβ, Fer-1 and LPS. **A** H&E staining of lung tissue after LPS challenge (n = 6). **B** Lung wet and dry weight ratio (W/D ratio) in mice challenged with LPS (n = 6). **C** Lung injury score in mice challenged with LPS (n = 6). **D** Levels of proinflammatory markers in lung tissue after LPS challenge (n = 6). **E** MDA levels in LPS-induced lung tissue (n = 6). **F** GSH levels in LPS-induced lung tissue (n = 6). **G** Fe^2+^ levels in LPS-induced mouse lung tissue (n = 6). *P < 0.05 vs. the CON group. #P < 0.05 vs. the LPS group. *NS* no significant

To confirm the ferroptosis targets of Metrnβ in vivo, mice treated with Fer-1 to inhibit ferroptosis. The increase in ALI induced by Metrnβ knockdown was abolished by Fer-1 treatment, as indicated by the reduced lung injury score and W/D ratio (Fig. 10a–c), improved inflammatory response (Fig. 10d), and decrease in ferroptosis (MDA, Fe2 + level, GSH level) (Fig. 10e–g) in the Fer-1 treatment group compared with the Ad-shMetrnβ group.

### SIRT1 knockout abolished the protective effects of Metrnβ overexpression in vivo

**Fig. 11:**
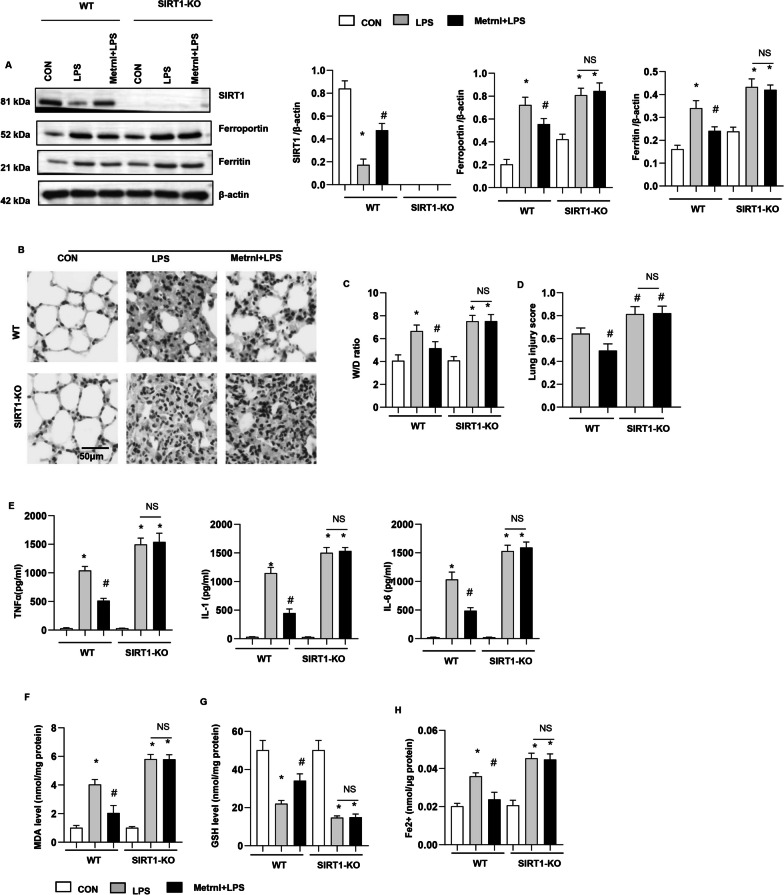
SIRT1 knockout abolished the protective effects of Metrnβ overexpression in vivo. WT or SIRT-1-knockout mice were treated with Ad-Metrnβ and/or LPS. **A** Protein levels of SIRT1, Ferroportin, and Ferritin in mice in each group (n = 4). **B** H&E staining of lung tissue in each group (n = 6). **C** Lung wet and dry weight ratios (W/D ratios) of mice in each group (n = 6). **D** Lung injury scores of mice in each group (n = 6). **E** Levels of proinflammatory markers in lung tissue in each group (n = 6). **F** MDA levels in lung tissue in each group (n = 6). **G** GSH levels in lung tissue in each group (n = 6). **H** Fe^2+^ levels in mouse lung tissue in each group (n = 6). *P < 0.05 vs. the CON/WT group. #P < 0.05 vs. the LPS/WT group. *NS* no significant

We showed that Metrnβ could activate the transcription of SIRT1 (Fig. 7). We then examined whether SIRT1 knockout could reverse the protective effects of Metrnβ in vivo. SIRT1 mice were subjected to LPS stimulation and transfected with Ad-Metrnβ to overexpress Metrnβ. As shown in Fig. 11a, the levels of Ferroportin and Ferritin were increased in SIRT1-KO mouse lung tissue. SIRT1 KO exacerbated LPS-induced lung injury and ferroptosis (Fig. 11b–g). Mice with both SIRT1 knockout and Metrnβ overexpression in lung tissue showed increased lung injury and ferroptosis compared with mice with only Metrnβ overexpression (Fig. 11b–g). These data indicate that Metrnβ regulates LPS-induced ALI and ferroptosis in lung tissue by targeting SIRT1.

## Discussion

ALI is an acute inflammatory reaction. Patients with severe disease can develop ARDS, which has a high incidence rate and leads to high mortality (Mokra 2020). ALI is caused by many factors, including direct lung injury such as infectious pneumonia and indirect (extrapulmonary) injury such as sepsis (Mokra and Kosutova 2015). Various forms of ALI share similar pathological processes, including alveolar capillary membrane dysfunction, enhanced inflammation and reduced alveolar fluid clearance, resulting in pulmonary edema, impaired gas exchange and persistent hypoxemia, leading to the death of patients (Mokra 2020). At present, although large numbers of studies have focused on developing specific drugs for ALI/ARDS, few results have been produced. Therefore, it is critical to further examine the pathological mechanism and potential therapeutic targets of ALI/ARDS. In this study, we confirmed the detrimental role of ferroptosis in LPS-induced ALI and found that Metrnβ could protects LPS-induced ALI and ferroptosis. Furthermore, Metrnβ directly affects AECs to inhibit inflammation, ferroptosis and dysfunction of epithelial cells, which ameliorate lung injury. We also observed the notable treatment efficacy of Metrnβ in lung tissue in protecting against LPS-induced ALI progression.

Ferroptosis, which is a newly discovered iron-dependent form of programmed cell death that was first reported in 2012, is distinct from apoptosis, necrosis, and autophagy (Chen et al. 2021). The main characteristics of ferroptosis are cell membrane integrity, cell volume reduction, an increase in mitochondrial membrane density, the reduction or even disappearance of mitochondrial cristae, mitochondrial membrane shrinkage and outer membrane rupture with normal nuclear size (Jiang et al. 2021). Ferroptosis is associated with intracellular iron accumulation, glutathione depletion, Gpx4 inactivation, and lipid peroxidation (Jiang et al. 2021). Recent studies have suggested that ferroptosis plays an important role in lung disease, pulmonary hypoxic injury, and pneumonia (Xu et al. 2021). Study have confirmed the role of ferroptosis-related genes in lipopolysaccharide-induced acute lung injury (Wang et al. 2023). In human coronavirus disease 2019 induced ALI, ferroptosis was also observed in human cardiac and lung tissue (Jacobs et al. 2020). Guo reported that inhibition of ferroptosis could alleive alveolar epithelial cells injury in ALI (Guo et al. 2022). Pan et al. also revealed that targeting ferroptosis was a promising therapeutic strategy for lung ischemia–reperfusion injury (Pan et al. 2022) Our study showed that in LPS-induced ALI, the iron concentration in lung tissue was significantly increased, ferroptosis-related Gpx4 and GSH were significantly decreased, lipid peroxidation was significantly increased, and the ferroptosis inhibitor Fer-1 alleviated these phenotypes and ameliorated LPS-induced lung injury. Metrnβ is a newly discovered secretory protein involved in glucose metabolism (Baht et al. 2020). A study found that a decrease in plasma Metrnβ was associated with insulin resistance in patients with type 2 diabetes (Wu et al. 2020). Celia Rupérez revealed that Metrnβ ameliorated cardiac dysfunction and cardiac hypertrophy in response to isoproterenol and ageing (Ushach et al. 2018; Ruperez et al. 2021). Ushach I reported that Metrnβ regulates inflammatory responses in macrophages. They also found that Metrnl−/− mice exhibited dysregulated cytokine production (Ushach et al. 2018). Most recently, Metrnβ was also reported to inhibit airway inflammation in house dust mite-induced allergic asthma (Gao et al. 2022). In this study, we first showed that Metrnβ could protect acute lung injury and AEC injury after LPS insult. Moreover, Metrnβ protected lung tissue from LPS-induced ferroptosis and inhibited ferroptosis in AECs independent of apoptosis inhibition. When we inhibited ferroptosis in vivo and in vitro, the protective effects of Metrnβ were counteracted. These results suggest that by inhibiting ferroptosis, Metrnβ plays a protective role in ALI.

Abnormal glutathione and GSH metabolism triggers ferroptosis. Gpx4 is an important enzyme for scavenging lipid oxygen free radicals (Li et al. 2020a; Xu et al. 2021). Gpx4 can reduce toxic lipid hydroperoxides to nontoxic lipid alcohols to inhibit ferroptosis (Xu et al. 2021). Cysteine is one of the main components of GSH synthesis. It can enter cells in two main ways, one of which depends on system xc- (Yu et al. 2021). System xc- is a heterodimer composed of solute carrier family 7 member 11 (SLC7A11) and solute carrier family 3 member 2 (SLC3A2), which is used for cysteine transport^6^. Studies have shown that P53 can inhibit the transcription of SLC7A11, resulting in reduced cysteine transport, reduced GSH synthesis and increased ferroptosis (Jiang et al. 2015; Chu et al. 2019). The SIRT1-p53 axis has been shown to regulate aging, cancer, and cellular reprogramming in many diseases (Ong and Ramasamy 2018; Song et al. 2019). Hu C et al. reported that Metrnβ could activate SIRT1 in cardiomyocytes and they showed that by activating cAMP/PKA mediated SIRT1 activation, Metrnβ attenuated doxorubicin-induced cardiotoxicity (Hu et al. 2020). However, in our study, we also showed that Metrnβ regulates ferroptosis in lung tissue and AECs by activating the SIRT1, subsequently inhibiting P53 and modulating SLC7A11 expression. We also confirmed that Metrnβ could directly activate the transcription of SIRT1 and thus mediated inhibition of P53-SLC7A11 pathway in lung tissue. This mechanism was fully confirmed by using SIRT1-KO mice. As we show that SIRT1-KO totally counteracted the protective effects of the protective effects of Metrnβ on ALI.

In summary, we found that Metrnβ was down-regulated in LPS-induced ALI and acted as a protective factor, inhibiting ferroptosis in AECs and could suppress lung injury. Furthermore, we first observed that Metrnβ regulates ferroptosis in ALI by modulating the SIRT1-P53- SLC7A11 pathway lung tissue.

### Limitation

There are some limitations of our study. we only used male mice to reduce the animal use. However sex might be an important factor in ALI, further study should aims to illustrate the function of Metrnβ in injury lung tissue in female ALI model in the future, to demonstrate whether sex play a role there. We used LPS injection to establish ALI mice model, whether Metrnβ can play the same effects in other types of ALI remains to be investigated. In addition, adenovirus delivery system was used in this study to overexpress or knockdown Metrnβ in lung tissue. The off-target effects of this system also need to be further explored.

## Data Availability

All the data will be available if requires from the corresponding author.
